# Supplementary material for: Using real-time modelling to inform the 2017 Ebola outbreak response in DR Congo
Source: Nat Commun. 2024 Jul 6;15:5667. doi: 10.1038/s41467-024-49888-5 (PMC11227569; doi:10.1038/s41467-024-49888-5)
Supplement: Supplementary file 1 — Supplementary Information [file 41467_2024_49888_MOESM1_ESM.pdf]

Supplementary Information for *Using real-time modelling to inform the 2017 Ebola outbreak response in DR Congo*

RN Thompson, WS Hart, M Keita, IS Fall, AS Gueye, D Chamla, M Mossoko, S Ahuka-Mundeke, J Nsio-Mbeta, T Jombart, J Polonsky

Supplementary Analysis 1: Effect of the probability distribution governing the daily number of cases

In the model underlying our main analyses, the number of cases occurring each day is drawn from a Poisson distribution. To incorporate the possibility that superspreading events can occur, an alternative choice is to assume that the number of cases each day is instead drawn from a more overdispersed distribution. In this supplementary analysis, we therefore present results that are analogous to those shown in Fig 2 in the main text, but with two different assumptions about the distribution governing the number of cases each day: i) Negative binomial distribution with dispersion parameter $k = 0.2$ (high chance of superspreading events); ii) Negative binomial distribution with dispersion parameter $k = 10$ (intermediate chance of superspreading events). The results shown in the main text correspond to $k \to \infty$.

To adapt the method in the main text to permit a negative binomial distributed number of cases each day, we note that the likelihood function for R given in equation (3) of the main text becomes

$$L(R) = \frac{1}{M_3} \prod_{t=2}^{49} \frac{\Gamma(k + I_t)}{I_t!\,\Gamma(k)} \left(\frac{R \sum_{s=1}^{t-1} I_{t-s} w_s}{k + R \sum_{s=1}^{t-1} I_{t-s} w_s} \right)^{I_t} \left(\frac{k}{k + R \sum_{s=1}^{t-1} I_{t-s} w_s} \right)^{k},$$

in which M_3 is a normalising constant (chosen so that $L(R)$ represents a valid probability density function).

Under this new model, the risk of withdrawing the ERT for a specific value of R is given by

$$\text{Risk of withdrawing ERT on day } t = 1 - \text{Prob(no cases from day } t \text{ onwards} \mid R),$$

$$= 1 - \prod_{j=t}^{\infty} \left(\frac{k}{k + R \sum_{s=1}^{j-1} I_{j-s} w_s} \right)^k .$$

As in the main text, the overall risk of withdrawing the ERT accounting for the full distributional estimate of R (i.e., $L(R)$) is

$$\text{Risk of withdrawing ERT on day } t$$

$$= 1 - \int_0^{\infty} \text{Prob(no cases from day } t \text{ onwards} \mid R) L(R) \, \mathrm{d}R.$$

Using this approach, we first estimated R in the absence of the ERT assuming that the dispersion parameter is $k = 0.2$ (Fig S2A – blue). The corresponding risk of withdrawing the ERT each day is shown in Fig S2B (blue). We then repeated this analysis, instead assuming that $k = 10$ (Fig S2A and S2B – red).

We found that, while the distributional estimate of R was sensitive to our choice of k, the resulting risk of withdrawing the ERT had a similar form in each scenario that we considered. Moreover, following a long period without cases, when the risk of withdrawing the ERT had fallen to a low value (so that a policy maker might consider withdrawing the ERT), the precise value of k did not have a substantial effect on the practical implications of the estimated ERT withdrawal risk. For example, if the ERT was withdrawn as soon as the corresponding risk fell below 0.05 (i.e., a less than 5% chance of future cases), then the withdrawal date would have been 25th June 2017 under an assumption that $k = 0.2$ and 22nd June 2017 under an assumption that $k = 10$ (as opposed to 21st June 2017 in our main analyses, which corresponds to $k \to \infty$).

Supplementary Analysis 2: Analysis of a larger outbreak

To demonstrate the generalisability of our modelling approach, we also applied it to data from a larger outbreak of EVD which took place in Équateur Province, DRC, from 5th April–2nd June 2018 (Fig S3A). In total, this outbreak comprised 54 cases. The ERT arrived on 8th May 2018 and was withdrawn on 24th July 2018, and we analysed the risk of withdrawing the ERT on each date following its arrival (again assuming that, if the ERT is withdrawn, the value of R reverts to its pre-ERT value).

Since this outbreak was larger than the one analysed in the main text, we were able to estimate the serial interval distribution using data from this specific outbreak rather than using a previously published estimate of the serial interval. In particular, following contact tracing, 41 realised serial intervals were recorded (these data represent all available observations of the serial interval recorded throughout the 2018 Équateur Province outbreak). We calculated the likelihood of these observed serial intervals being realised under a discretised gamma distributed serial interval with mean μ and standard deviation σ. Discretisation of the serial interval was performed using equation (2) in the main text. The resulting likelihood profile is shown in Fig S3B. We used the maximum likelihood estimate ($\mu = 19.46$, $\sigma = 6.08$; see Fig S3C) in our subsequent estimation of R (in the absence of the ERT) and the risk of withdrawing the ERT.

The value of R in the absence of the ERT was estimated based on the incidence data prior to the arrival of the ERT (from 5th April to 7th May 2018, adapting equation (3) in the main text to cover this date range). The resulting distributional estimate of R is shown in Fig S3D (blue line). Then, we computed the risk of withdrawing the ERT each day based on the entire distributional estimate of R (using equation (5) in the main text, as in our main analysis), shown in Fig S3E (blue line).

In addition to showing the distributional R estimate and the risk of withdrawing the ERT for this outbreak-specific serial interval, we also present the analogous results using the serial interval considered in our main analyses (blue dotted lines in Figs S3D and S3E; the original serial interval distribution is shown in Fig S1). While the outbreak-specific serial interval has a larger mean than the one used in our main analyses, its lower standard deviation means that very long serial intervals are unlikely (*cf.* Fig S3C and Fig S1). As a result, the risk of withdrawing the ERT was lower after a long period without cases when the outbreak-specific serial interval was used compared to when the serial interval from our main analyses was used. In particular, by the date on which the ERT was actually withdrawn, additional cases in the absence of the ERT were very unlikely. Since the results in Fig S3E are not identical when different serial interval distributions are used, we contend that, when available, observed serial intervals from the specific outbreak being analysed should be considered when estimating R or calculating the risk of withdrawing the ERT.

Supplementary Analysis 3: Effect of underreporting

In our main analyses of the EVD outbreak in Likati Health Zone, DRC, we assumed that all cases were recorded. While this was likely a reasonable assumption for that outbreak, due to its small size and the case finding activities that were undertaken, underreporting of cases can beset epidemiological assessments during outbreaks of many pathogens. We therefore conducted a supplementary analysis to investigate the impact of underreporting on estimates of the risk of withdrawing the ERT.

Specifically, due to its larger size (and therefore higher risk of underreporting), we considered the Équateur Province EVD outbreak analysed in Supplementary Analysis 2. Using the maximum likelihood serial interval distribution found in that analysis (Fig S3C), we

estimated R in two different time periods: i) From 5th April–7th May 2018 (i.e., in the absence of the ERT, as in the blue line in Fig S3D), and ii) From 8th May–23rd July 2018 (i.e., when the ERT was present). Using these two distributional estimates of R, we calculated the relative force of infection on each day (which we denote $p(t)$) based on the observed cases in the outbreak, using the formula

$$p(t) = \frac{1}{M_4} \int_0^\infty R \sum_{s=1}^{t-1} I_{t-s} w_s \, L(R) \mathrm{d}R = \frac{1}{M_4} \mathbb{E}(R) \sum_{s=1}^{t-1} I_{t-s} w_s.$$

In this expression, the relevant likelihood function $L(R)$ is used (as is the relevant expected value of R, $\mathbb{E}(R)$), depending on whether or not the ERT was in place on day t (in other words, the distributional R estimate in the absence of the ERT was used for days t lying in the period from 5th April–7th May 2018, and the equivalent distributional estimate in the presence of the ERT was used for days t from 8th May–23rd July 2018). The variable M_4 is a normalising constant, so that the expression above is a valid probability mass function. The function $p(t)$ thereby provides an approximation of the relative risk of a missed case each day, based on the dates of reported cases. The resulting distribution is shown in Fig S4A.

We then conducted analyses in which we assumed that m cases had been missed (we repeated this analysis for values of m between one and five; this corresponds to increasing the size of the outbreak above the number of reported cases by between 1.9% and 9.3%). Specifically, we sampled the days on which those missed cases occurred from the distribution characterising the relative force of infection on each day, $p(t)$ (we assumed that these samples were independent of each other). We then re-estimated the posterior distribution for R in the absence of the ERT (i.e., $L(R)$) using the new incidence data (including the unreported cases) and inferred the risk of withdrawing the ERT each day following its arrival. We repeated this analysis (separately for each value of m) 10,000 times, averaging the results

to obtain the risk of withdrawing the ERT each day based on different possible dates on which cases may have gone unreported (Fig S4B).

We found that, while the risk of withdrawing the ERT was highest if we assumed a large number of unreported cases, the effect of underreporting on policy decisions may be less than might be expected. For example, assuming that two cases were not reported and the ERT can be withdrawn when the estimated ERT withdrawal risk falls below 0.01 (corresponding to a 1% chance of further cases occurring), the theoretical ERT withdrawal date was 24th July 2018 (green line in Fig S4B). On the other hand, if five cases were not reported (corresponding to increasing the outbreak size by 9.6% compared to the number of observed cases), then the analogous theoretical ERT withdrawal date was five days later on 29th July 2018 (purple line in Fig S4B). Despite the fact that the difference between these two dates might be considered to be relatively small, our results indicate that different assumed numbers of unreported cases can correspond to different theoretical ERT withdrawal dates. A policy maker who is risk averse may choose to base their decision about when to withdraw the ERT on analyses undertaken assuming a high level of underreporting.

Supplementary Text 1: Additional details about the method used to estimate R

In all our analyses, we estimated the value of R in the absence of the ERT by calculating the likelihood of observing the incidence data up to (and including) the day before the arrival of the ERT. It should be noted that, when we analysed the data from the 2017 EVD outbreak in Likati Health Zone, it was possible to estimate R precisely despite the relatively small number of cases (eight cases occurred in total in that outbreak). This is because we assumed that R took a constant value throughout the period from the first case in the outbreak to the

arrival of the ERT. The information available with which to estimate R accumulated across this time period. Had we instead assumed that R varied temporally before the ERT was deployed, it would likely have been impossible to monitor variations in R from such little data.

We provide more details about our calculation of R in the absence of the ERT (as undertaken in the main text) here. First, we note that, for a fixed value of R, the probability of observing the incidence data that arose following the index case and prior to the arrival of the ERT during the 2017 EVD outbreak in Likati Health Zone (i.e., the incidence data $\{I_t\}_{t=2}^{49}$) is given by

$$\mathbb{P}(\{I_t\}_{t=2}^{49} \mid R) = \prod_{t=2}^{49} \frac{\left(\mathbb{E}(I_t \mid R)\right)^{I_t} \exp\left(-\mathbb{E}(I_t \mid R)\right)}{I_t!}.$$

This corresponds to the number of cases each day being drawn from a Poisson distribution, in which the mean number of cases on day t depends on the numbers of cases on previous days,

$$\mathbb{E}(I_t \mid R) = R \sum_{s=1}^{t-1} I_{t-s} w_s.$$

The likelihood function for R, $L(R)$, represents the relative likelihood of different values of R. In other words,

$$L(R) = \frac{\mathbb{P}(\{I_t\}_{t=2}^{49} \mid R)}{M_1}.$$

In this expression, the normalisation factor M_1 is chosen so that $\int_0^\infty L(R)\,\mathrm{d}R = 1$. Consequently, $L(R)$ is a valid probability density function characterising the relative

likelihood of different values of R leading to the incidence data observed from $t = 2$ to $t = 49$.

We note that $L(R)$ corresponds to the posterior estimate of R assuming an uninformative (uniform) prior. To instead include an informative prior ($\rho(R)$, say), the formula for $L(R)$ should be multiplied by $\rho(R)$, and the resulting expression renormalised. In other words, the posterior for R would more generally be $\rho(R)L(R)/M_5$, in which M_5 is chosen so that the posterior is a valid probability density function (i.e., $M_5 = \int_0^\infty \rho(R)L(R)\,\mathrm{d}R$).

Supplementary Figures

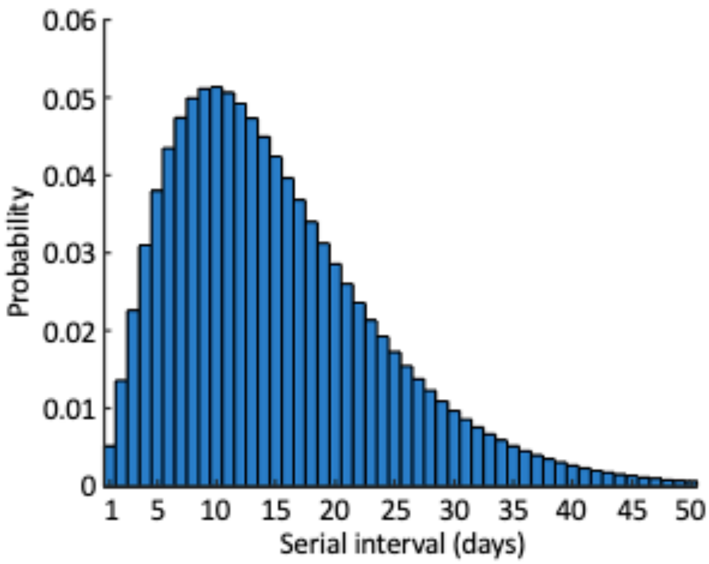

Figure S1. The discrete serial interval distribution assumed in our main analyses. To obtain this distribution, the continuous serial interval was assumed to be a gamma distribution with mean 15.3 days and standard deviation 9.3 days [1]. The continuous distribution was then discretised using the method from [2] (see equation (2) in the main text).

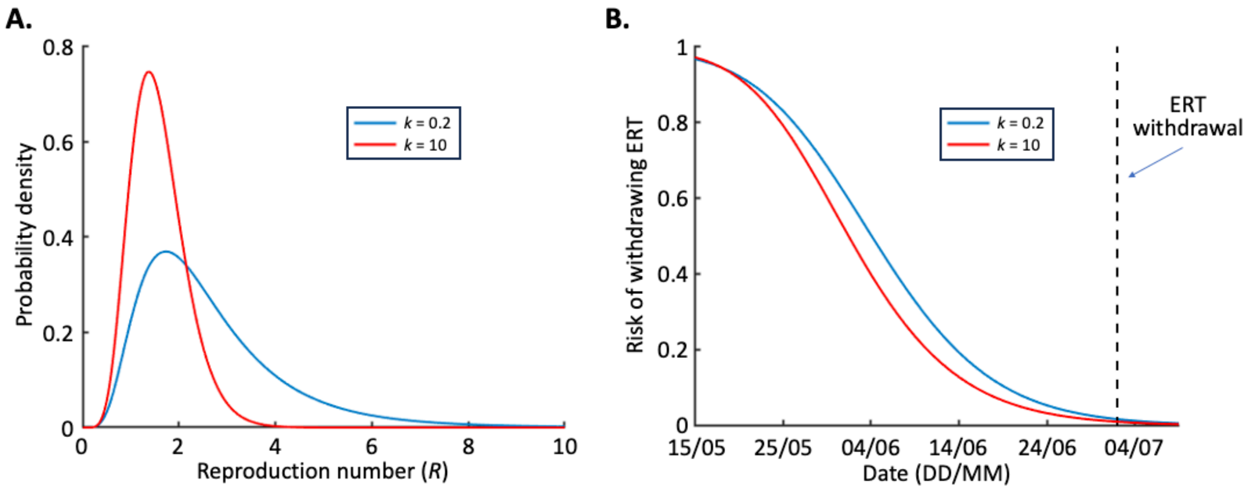

Figure S2. Robustness of results to assumed probability distribution governing the number of cases each day. Analogous results to those shown in Fig 2 of the main text, but instead assuming that the daily number of cases is drawn from a negative binomial distribution (with dispersion parameter k). A. The estimated value of R prior to the arrival of the ERT, shown for values of $k = 0.2$ (blue; corresponding to a high chance of superspreading events) and $k = 10$ (red; corresponding to an intermediate chance of superspreading events). B. The risk of withdrawing the ERT (the probability of future cases occurring if the ERT is withdrawn on each date on the x-axis, based on the distributional estimates of R shown in panel A).

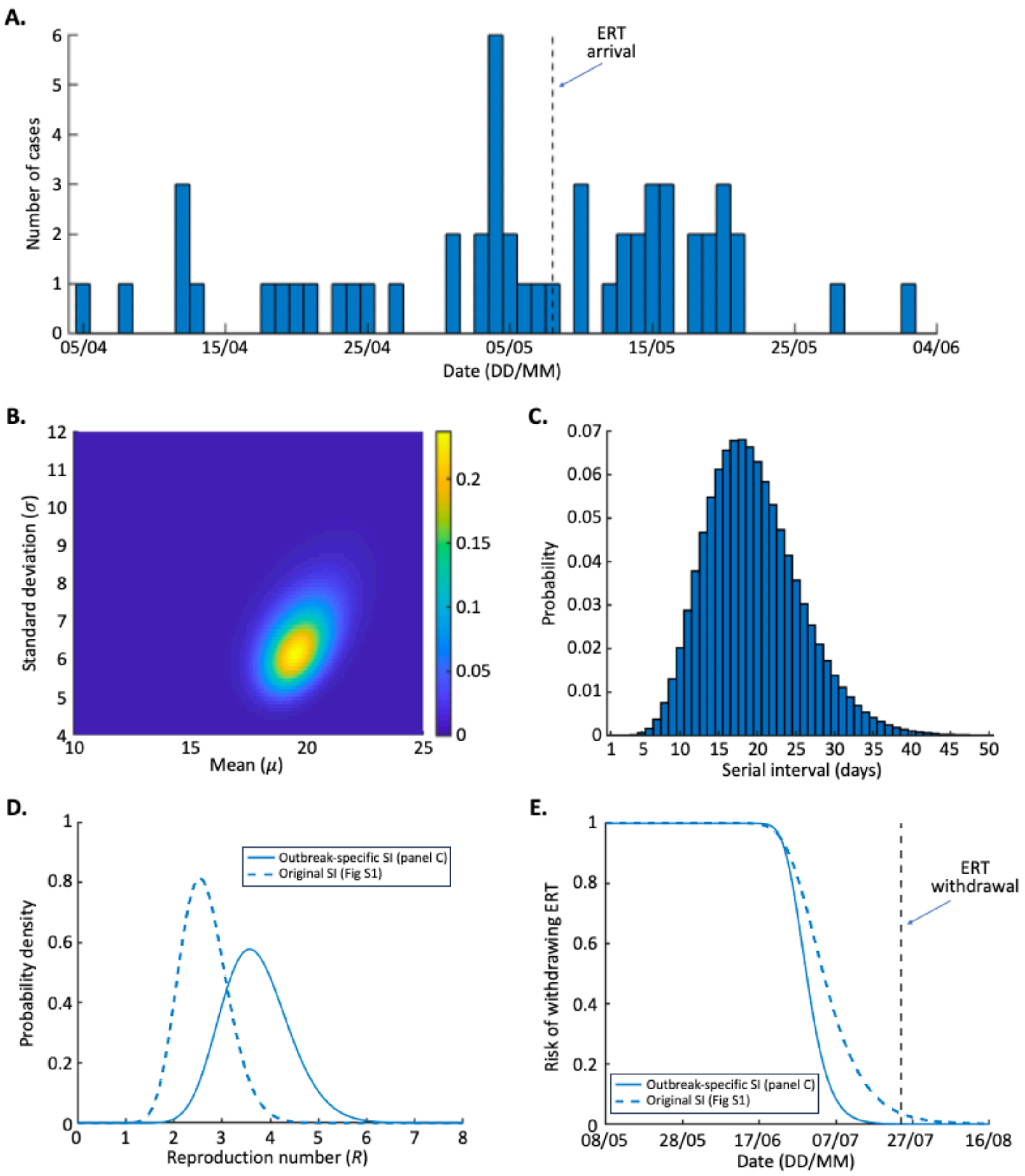

Figure S3. Application of our modelling approach to data from the EVD outbreak in Équateur Province, DRC, from 5th April to 2nd June 2018. A. Daily numbers of cases occurring during the outbreak (blue) and the date of the ERT arrival (8th May 2018; black dotted); B. Likelihood of 41 serial intervals reported during the outbreak under a discretised gamma distributed serial interval with mean μ and standard deviation σ. C. The maximum likelihood serial interval ($\mu = 19.46$, $\sigma = 6.08$) based on the likelihood surface shown in panel B. D. The estimated value of R prior to the arrival of the ERT, calculated using equation (3) in the main text (adapted to cover the period from 5th May–7th April 2018) with either the outbreak-specific serial interval distribution in panel C (blue) or the original EVD serial interval distribution in Fig S1 (blue dashed). E. The risk of withdrawing the ERT as calculated each day (equation (5) in the main text; i.e., the probability of future cases occurring if the ERT is withdrawn on each date on the x-axis, based on the distributional estimates of R in panel D with the corresponding serial interval distributions). The actual date of ERT withdrawal is shown as a black dashed line.

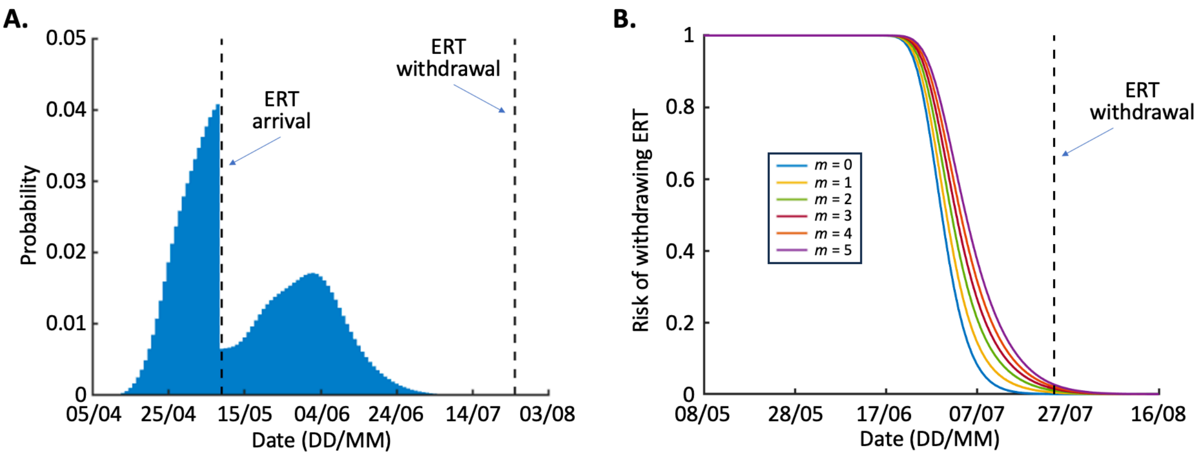

Figure S4. Incorporation of underreporting into estimates of the risk of withdrawing the ERT. A. Conditional on an unreported case occurring, the probability $p(t)$ that it arose on each day of the 2018 EVD outbreak in Équateur Province, DRC, based on the dates of the reported cases (Fig S3A), the estimated values of R with and without the ERT, and the serial interval distribution (Fig S3C). B. The risk of withdrawing the ERT as calculated each day (i.e., the probability of future cases occurring if the ERT is withdrawn on each date on the x-axis) using the serial interval distribution in Fig S3C, for different numbers of missed cases (m). These results were obtained by sampling the date of each missed case independently from the probability distribution shown in panel A and then re-estimating both R in the absence of the ERT and the risk of withdrawing the ERT. The range of different possible dates on which cases were missed was considered by repeating this calculation 10,000 times and then averaging the results (separately for each value of m).

References

1. Van Kerkhove MD, Bento AI, Mills HL, Ferguson NM, Donnelly CA. A review of epidemiological parameters from Ebola outbreaks to inform early public health decision-making. Sci Data. 2015;2: 150019.

2. Cori A, Ferguson NM, Fraser C, Cauchemez S. A new framework and software to estimate time-varying reproduction numbers during epidemics. Am J Epidemiol. 2013;178: 1505–12.
