# Supplementary material for: Using real-time modelling to inform the 2017 Ebola outbreak response in DR Congo
Source: Nat Commun. 2024 Jul 6;15:5667. doi: 10.1038/s41467-024-49888-5 (PMC11227569; doi:10.1038/s41467-024-49888-5)
Supplement: Supplementary file 3 — Reporting Summary [file 41467_2024_49888_MOESM3_ESM.pdf]

nature portfolio

Corresponding author(s): Robin N Thompson

Last updated by author(s): Apr 16, 2024

Reporting Summary

Nature Portfolio wishes to improve the reproducibility of the work that we publish. This form provides structure for consistency and transparency in reporting. For further information on Nature Portfolio policies, see our Editorial Policies and the Editorial Policy Checklist.

Statistics

For all statistical analyses, confirm that the following items are present in the figure legend, table legend, main text, or Methods section.

n/a	Confirmed	
☒	☐	The exact sample size (*n*) for each experimental group/condition, given as a discrete number and unit of measurement
☒	☐	A statement on whether measurements were taken from distinct samples or whether the same sample was measured repeatedly
☒	☐	The statistical test(s) used AND whether they are one- or two-sided<br>*Only common tests should be described solely by name; describe more complex techniques in the Methods section.*
☒	☐	A description of all covariates tested
☒	☐	A description of any assumptions or corrections, such as tests of normality and adjustment for multiple comparisons
☐	☒	A full description of the statistical parameters including central tendency (e.g. means) or other basic estimates (e.g. regression coefficient) AND variation (e.g. standard deviation) or associated estimates of uncertainty (e.g. confidence intervals)
☒	☐	For null hypothesis testing, the test statistic (e.g. *F*, *t*, *r*) with confidence intervals, effect sizes, degrees of freedom and *P* value noted<br>*Give P values as exact values whenever suitable.*
☐	☒	For Bayesian analysis, information on the choice of priors and Markov chain Monte Carlo settings
☒	☐	For hierarchical and complex designs, identification of the appropriate level for tests and full reporting of outcomes
☒	☐	Estimates of effect sizes (e.g. Cohen's *d*, Pearson's *r*), indicating how they were calculated

Our web collection on statistics for biologists contains articles on many of the points above.

Software and code

Policy information about availability of computer code

Data collection	No software was used for data collection in this study.
Data analysis	The computing code that was written and used to perform the analyses in this article was written in Matlab (compatible with version 2022a).

For manuscripts utilizing custom algorithms or software that are central to the research but not yet described in published literature, software must be made available to editors and reviewers. We strongly encourage code deposition in a community repository (e.g. GitHub). See the Nature Portfolio guidelines for submitting code & software for further information.

Data

Policy information about availability of data

All manuscripts must include a data availability statement. This statement should provide the following information, where applicable:

- Accession codes, unique identifiers, or web links for publicly available datasets
- A description of any restrictions on data availability
- For clinical datasets or third party data, please ensure that the statement adheres to our policy

The data and computing code used to perform the analyses in this article are available in the following GitHub repository: https://github.com/robin-thompson/EbolaReponseTeam. All code was written in Matlab (compatible with version 2022a). No restrictions on data availability exist.

Research involving human participants, their data, or biological material

Policy information about studies with human participants or human data. See also policy information about sex, gender (identity/presentation), and sexual orientation and race, ethnicity and racism.

Reporting on sex and gender	Sex- and gender-based analyses were not relevant to our study. Participants were not recruited to the study; instead, all available outbreak data were analysed (these data were reported as number of cases per day, irrespective of sex or gender).
Reporting on race, ethnicity, or other socially relevant groupings	Analyses of race, ethnicity or social groupings were not relevant to our study. Participants were not recruited to the study; instead, all available outbreak data were analysed (these data were reported as number of cases per day; data on social groupings were not collected).
Population characteristics	Analyses of population characteristics were not relevant to our study. Participants were not recruited to the study; instead, all available outbreak data were analysed (these data were reported as number of cases per day).
Recruitment	Participants were not recruited specifically for the purpose of this study. Data were collected as part of the DRC government-led outbreak response.
Ethics oversight	Data were collected as part of the DRC government-led outbreak response. Ethics approval was granted by the ethics committee at the Kinshasa School of Public Health - the approval number is ESP/CE/03/2021.

Note that full information on the approval of the study protocol must also be provided in the manuscript.

Field-specific reporting

Please select the one below that is the best fit for your research. If you are not sure, read the appropriate sections before making your selection.

☒ Life sciences ☐ Behavioural & social sciences ☐ Ecological, evolutionary & environmental sciences

For a reference copy of the document with all sections, see nature.com/documents/nr-reporting-summary-flat.pdf

Life sciences study design

All studies must disclose on these points even when the disclosure is negative.

Sample size	This is not relevant to our study; no sample size calculation was performed because analyses were based on all available outbreak data (experiments were not undertaken for the purpose of our study).
Data exclusions	No data were excluded from the analyses.
Replication	The computational findings were reproduced by two of the co-authors on this study (RNT and WSH) to verify the results.
Randomization	This is not relevant to our study; no allocation of participants into groups was required for the analyses undertaken.
Blinding	This is not relevant to our study; no allocation of participants into groups was required for the analyses undertaken.

Reporting for specific materials, systems and methods

We require information from authors about some types of materials, experimental systems and methods used in many studies. Here, indicate whether each material, system or method listed is relevant to your study. If you are not sure if a list item applies to your research, read the appropriate section before selecting a response.

Materials & experimental systems

n/a	Involved in the study	
☒	☐	Antibodies
☒	☐	Eukaryotic cell lines
☒	☐	Palaeontology and archaeology
☒	☐	Animals and other organisms
☒	☐	Clinical data
☒	☐	Dual use research of concern
☒	☐	Plants

Methods

n/a	Involved in the study	
☒	☐	ChIP-seq
☒	☐	Flow cytometry
☒	☐	MRI-based neuroimaging

Plants

Seed stocks	No seed stocks were used.
Novel plant genotypes	No novel plant genotypes were produced.
Authentication	Not applicable.
